# Effects of Moderate Strength Cold Air Exposure on Blood Pressure and Biochemical Indicators among Cardiovascular and Cerebrovascular Patients

**DOI:** 10.3390/ijerph110302472

**Published:** 2014-02-27

**Authors:** Xiakun Zhang, Shuyu Zhang, Chunling Wang, Baojian Wang, Pinwen Guo

**Affiliations:** 1 School of Atmospheric Sciences, Nanjing University of Information Sciences and Technology, 219 Ningliu Road, Nanjing 210044, China; E-Mails: zxk668@126.com (X.Z.); guo@nuist.edu.cn (P.G.); 2 Key Laboratory of Arid Climatic Change and Reducing Disaster of Gansu Province, Lanzhou Institute of Arid Meteorology, China Meteorological Administration, 2070 Donggang East Road, Lanzhou 730020, China; 3 School of Applied Meteorology, Nanjing University of Information Sciences and Technology, 219 Ningliu Road, Nanjing 210044, China; E-Mail: wangchunling668@126.com; 4 Lanzhou Central Meteorological Observatory, 2070 Donggang East Road, Lanzhou 730020, China; E-Mail: baojianwang@126.com

**Keywords:** cold air, catecholamine, myoglobin, endothefin-1, cardiovascular disease

## Abstract

The effects of cold air on cardiovascular and cerebrovascular diseases were investigated in an experimental study examining blood pressure and biochemical indicators. Zhangye, a city in Gansu Province, China, was selected as the experimental site. Health screening and blood tests were conducted, and finally, 30 cardiovascular disease patients and 40 healthy subjects were recruited. The experiment was performed during a cold event during 27–28 April 2013. Blood pressure, catecholamine, angiotensin II (ANG-II), cardiac troponin I (cTnI), muscle myoglobin (Mb) and endothefin-1 (ET-1) levels of the subjects were evaluated 1 day before, during the 2nd day of the cold exposure and 1 day after the cold air exposure. Our results suggest that cold air exposure increases blood pressure in cardiovascular disease patients and healthy subjects via the sympathetic nervous system (SNS) that is activated first and which augments ANG-II levels accelerating the release of the norepinephrine and stimulates the renin-angiotensin system (RAS). The combined effect of these factors leads to a rise in blood pressure. In addition, cold air exposure can cause significant metabolism and secretion of Mb, cTnI and ET-1 in subjects; taking the patient group as an example, ET-1 was 202.7 ng/L during the cold air exposure, increased 58 ng/L compared with before the cold air exposure, Mb and cTnI levels remained relatively high (2,219.5 ng/L and 613.2 ng/L, increased 642.1 ng/L and 306.5 ng/L compared with before the cold air exposure, respectively) 1-day after the cold exposure. This showed that cold air can cause damage to patients’ heart cells, and the damage cannot be rapidly repaired. Some of the responses related to the biochemical markers indicated that cold exposure increased cardiovascular strain and possible myocardial injury.

## 1. Introduction

With the worldwide concern and study of cardiovascular disease caused by low temperatures, cold air exposure has been acknowledged as an important weather risk factor, which affects the increase in morbidity and mortality of cardiovascular disease [1,2,3,4,5,6,7,8,9,10,11,12]. A study by the World Health Organization showed that incidence of hypertensive disorders and related cardiovascular diseases in colder northern China is significantly higher than that in warmer southern China [13]. Cold weather can aggravate hypertension disease, and increase the incidence of hypertension-associated cardiovascular diseases such as stroke and myocardial infarction [3,14,15]. Several epidemiological studies demonstrated that cold air stimulation can result in blood pressure increase [16,17,18,19,20]; the mechanism of impact has been previously studied in animal experiments [21,22,23]. We wanted to determine whether a similar mechanism was present in cardiovascular disease patients. In this paper, cold air of moderate intensity in Zhangye City of Gansu Province in northern China was used as the experimental example to study. Zhangye City has a complex climate, changeable weather, and large temperature differences. It is the choke point through which cold air from China’s northwest must pass to the southeast. Each year approximately 95% of the cold air affecting China passes through Gansu Province. Adverse weather conditions have a strong impact on the local residents’ lives and health, especially cardiovascular disease patients [24,25].

In our studies, the effects of moderate-intensity cold air observed in April 2013 in cardiovascular and cerebrovascular disease patients was evaluated based on a previous studies in healthy and hypertensive rats. Blood pressure, catecholamine, angiotensin II (ANG-II), cardiac troponin I (cTnI), myoglobin (Mb), and endothelin-1(ET-1) levels of both cardiovascular disease patients and healthy subjects were measured before, during, and after cold air exposure with the aim of investigating the effects on cardiovascular and cerebrovascular diseases induced by changes in catecholamine.

## 2. Experimental Section

### 2.1. Study Site

Zhangye City (38.9°N, 100.5°E), Gansu Province, was chosen as the study site. Zhangye is a city in northwestern China, which has a typically temperate, continental and arid climate with four distinct seasons including long, cold winters and short, mild summers. The mean temperature is 7.3 °C; total annual precipitation averages 130.4 mm; and the mean wind speed is 2.0 m/s. There is good air cleanliness with no chemical pollution, and air quality meets the set ambient air standards. With control of middle and high-latitude westerly circulation and influence by arctic cold air masses, there is also highly variable weather, and large temperature variations. Cold air pouring south must pass through this location.

### 2.2. Study Subjects and Data Acquisition

A random cluster sampling method was used, and the Zhangye City People’s Hospital was selected as a monitoring point. Health records of residents between the ages of 40 and 70 years living within 1,000 m of the monitoring point were examined. Cardiovascular and cerebrovascular disease patients without organic disease were selected according to health screening and blood tests. Before the on-site study, 70 volunteers of cardiovascular disease patients, none with alcohol addiction, and none having taken medication for at least 3 days, were selected to form the patient group. At the same time, 70 healthy subjects chosen on the same inclusion criteria were selected to form the control group (sound in body and mind, none having any disease recently). 

In the period of 26–29 April 2013, volunteers participating in the experiment were recruited in the Zhangye City People’s Hospital every morning. A questionnaire survey was firstly administered by the study groups. The questionnaire included questions on physical condition, diet, medication, activities, *etc.* The purpose of the questionnaire was to rule out confounding factors and ensure the same exposure history between the patient and control groups. Those who were late or absent in blood sampling or blood pressure measurement, taking any drugs, suffering from mental stimulation, or suffering from influenza or any other diseases during the experiment was abandoned. Then a variety of measurements were conducted in accordance with the experimental requirements. The final data came from 30 patients (16 males, 14 females) with cardiovascular or cerebrovascular disease and 40 healthy controls (24 males, 16 females) who strictly complied with the experimental requirements during the whole period of the experiment. Cardiovascular and cerebrovascular diseases mainly included thrombosis, stroke, myocardial infarction, coronary heart disease and high blood pressure.

The study was reviewed and approved by the Medical Ethics Committee of Zhangye City People’s Hospital before the experiment began. All the volunteers provided their written informed consent to participate in this experiment. This consent procedure was approved by the Medical Ethics Committee of Zhangye City People’s Hospital and all the written informed consent was archived by the Committee.

Determination: The measurement indicators were blood pressure, catecholamine, ANG-II, cTnI, Mb, and ET-1 levels.

Sample collection: Five mL samples of fasting venous blood were collected from each volunteer 1 day before cold air exposure (8:00–8:30, 26 April), during the 2nd day of the cold exposure (at minimum temperature, 7:00–7:30, 28 April), and 1 day after the cold air exposure (8:00–8:30, 29 April). Samples were collected in vacuum blood collection tubes without anticoagulant. After centrifugation at 3,000 rpm, the serum was frozen at −80 °C. Blood pressure measurement for every participant volunteers, including diastolic and systolic, performed in every morning during the experiment. Mercury sphygmomanometer was used for the measurement.

Determination: The enzyme-linked immunosorbent assay (ELISA) double antibody sandwich method was used to determine catecholamine level. The steps were as follows: (1) a microtiter plate was coated with purified antibody to make a solid-phase antibody; (2) a test sample and the enzyme reagent were added to form an antibody-antigen-enzyme-antibody complex; (3) a chromogenic agent was added after washing; (4) the absorbance was measured at 450 nm; and (5) the concentration of the test sample was calculated.

The ELISA kit was produced by an American R&D company (Minneapolis, MN, USA) and packaged by Xi’an Kehao Biological Engineering Co., Ltd. (Xi’an, China), and the microplate reader was produced by the Tecan Company (Grödig, Austria). Detection was performed by the Medical Research Center, Lanzhou University.

Meteorological data: The crowd experimental study in Zhangye City, Gansu Province was conducted on 27–28 April 2013 during the onset of cold air. Cold air weather data including temperature, atmospheric pressure, and other hourly monitoring data and the weather forecast for cold air activity were provided by the Lanzhou Central Meteorological Observatory. Cold air weather event was determined according to China’s Cold Air Level National Standard (GB/T20484-2006) developed by the Central Meteorological Observatory in 2006 [26]. 

### 2.3. Statistical Methods

SPSS13.0 software (SPSS Inc., Chicago, IL, USA) was used for statistical analysis of data. The chi-square test was used to compare the sex and age composition of the patient and control groups. A randomized block design two-factor variance analysis was used for different times, groups, and gender rheology data; the Mann-Whitney U test for comparing two independent samples was used to compare between subjects with or without a cardiovascular or cerebrovascular diseases; the one-way ANOVA was used to compare the indicators before, during, and after cold air exposure; and Wilcoxon two-related sample tests were used to compare the patient and control groups. These test standards were based on a = 0.05.

## 3. Results and Discussion

### 3.1. Analysis of Changes in Cold Air

Table 1 showed that in Zhangye City, the minimum temperature on 26 April 2013 was 16.2 °C and that on 28 April it was 8.8 °C. Thus, the minimum temperature dropped by 7.4 °C in 48 h. China’s national cold air level standards (GB/T20484-2006) [26] confirms that cold air showing a daily minimum temperature drop greater than or equal to 6 °C but less than 8 °C is moderate strength cold air weather event. This cold air weather event influenced Zhangye from 6:00, 27 April. Minimum temperature was at 7:00 on the 28th, and the cold air activity ended at 23:00 on the 28th.

**Table 1:** The basic meteorological data of the cold air event in Zhangye City, April 2013 (°C).

Variables	26th	27th	28th	29th
Tmax_24_	26.1	19.4	16.4	26.5
Tmin_24_	16.2	14.9	8.8	10.4
ΔTmin_48_	7.4			

Notes: Tmax_24_ denotes daily maximum temperature; Tmin24 denotes daily minimum temperature, and ΔTmin_48_ denotes minimum temperature difference in 48 h.

### 3.2. Analysis of the Basic Situation of the Experimental Groups

According to the requirements, the basic description of the subjects participating in the moderate-intensity cold experiment on 26–29 April was as follows (Table 2): the patient group comprised 30 cases, with a sex ratio of 1:1, an average age of 59 years, including six cases of cerebral thrombosis, two cases of cerebral hemorrhage, 12 cases of coronary heart disease, and 10 cases of hypertension. The control group comprised 40 cases, with a sex ratio of 3:2 (male: female) and an average age of 55 years. The difference in sex and age composition between the patient and control groups was not statistically significant.

**Table 2:** Gender and age compositions of the patient and control groups.

Group	Cases	Gender/n (%)			Age Composition/n (%)			
		Male	Female		40 to	50 to	60 to 70	*x* ± s
Control	40	24 (60.0)	16 (40.0)		11 (27.5)	14 (35.0)	15 (37.5)	55 ± 9.8
Patient	30	15 (50.0)	15 (50.0)		9 (30.0)	9 (30.0)	12 (40.0)	59 ± 10.0
Total	70	39 (57.1)	31 (42.9)		20 (28.6)	23 (32.8)	27 (38.6)	57 ± 9.6

### 3.3. Results of Catecholamine Detection

#### 3.3.1. Comparison of Results of the Same Group at Different Times

As shown in Table 3, dopamine (DA) levels in both the patient and control groups before, during, and after cold air exposure decreased, and differences were significant (*p* < 0.05). Epinephrine (E) and norepinephrine (NE) levels increased slightly, but with no significant difference. The three indicators of the patient and control groups were compared. Except for E during the cold air exposure, other indicators of the patient group were slightly higher than those of the control group, but not significantly higher.

#### 3.3.2. Analysis of Catecholamine Trend during the Cold Air Event

As shown in Table 3, when comparing catecholamine levels before, during, and after cold air exposure patient groups, DA levels had declines of 3.5% and 91.03% compared with before cold air exposure groups and showed a negative trend; the smallest change was in E levels, representing increases of 0.17% and 0.51%; and the increases in NE levels were 93.3% and 128.93%; both NE and E showed a positive growth trend. During the cold air exposure, DA, E and NE levels in the control group showed varying degrees of elevation relative to the time before cold air exposure and the increases were 23.16%, 19.72% and 61.59%, respectively. After the cold air exposure, DA levels in the control group dropped to levels lower than those before and during cold air exposure, and declines were 81.47%, 84.95%, respectively. E and NE levels dropped but were higher than the levels before cold exposure. 

**Table 3:** Average catecholamine levels in the patient and control groups during a cold air event (mean ± standard deviation, ng/L).

Time	Patient Group (Cardiovascular Disease Patients)			Control Group (Healthy People)		
	DA	NE	E	DA	NE	E
Before cold air exposure	716.6 ± 72.1	158.7 ± 23.3	77.3 ± 9.6	345.0 ± 28.4	172.9 ± 18.4	67.9 ± 8.6
During cold air exposure	691.5 ± 58.7	306.9 ± 32.2	78.7 ± 8.7	424.9 ± 23.7 *	279.4 ± 25.1	81.3 ± 10.7
After cold air exposure	64.2 ± 11.5 *^,#^	363.4 ± 38.5 *^,#^	81.3 ± 10.9	64.0 ± 7.7 *^,#^	267.7 ± 21.7 *	78.0 ± 5.7 *
Significant test	χ^2^ = 8.132	χ^2^ = 9.013	χ^2^ = 1.176	χ^2^ = 12.362	χ^2^ = 0.449	χ^2^ = 3.956
(Mann-Whitney U)	*p* = 0.015	*p* = 0.132	*p* = 0.553	*p* = 0.001	*p* = 0.793	*p* = 0.153

Notes: * compared with the indicators before the cold air exposure, *p* < 0.05; **^#^** compared with the indicators during the cold air exposure, *p* < 0.05; DA: dopamine; E: epinephrine; NE: norepinephrine.

### 3.4. Analysis of the Myocardium and Vascular Protein Test Results

#### 3.4.1. Mb Test Results Analysis

**Figure 1:**
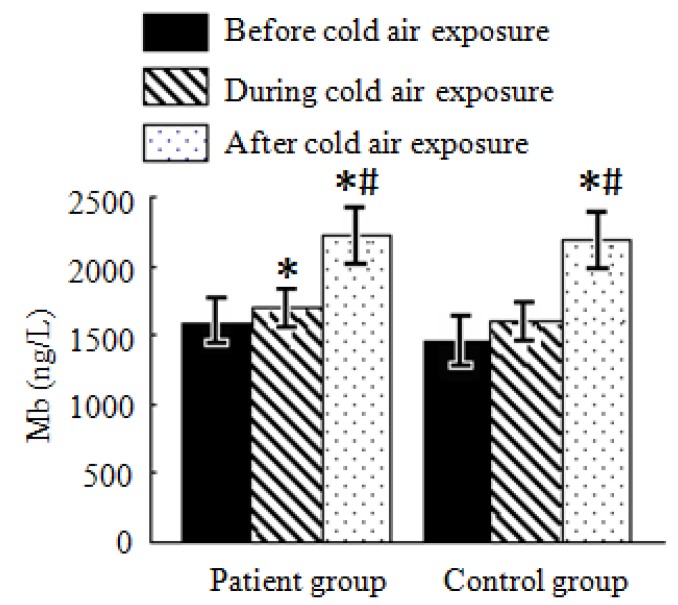
Average Mb levels in the patient and control groups during a cold air event.

Notes: * compared with the indicators before the cold air exposure, *p* < 0.05; **#** comparison with the indicators during the cold air exposure, *p* < 0.05; Mb: myoglobin; Error bars: standard deviation.

As shown in Figure 1, Mb levels in both the patient and control groups before, during, and after cold air exposure increased and differences were significant (*p* < 0.05). Compared with Mb levels before cold air exposure, it respectively elevated by 124.5 ng/L and 644.1 ng/L in during and after cold air exposure in the patient groups while it respectively elevated by 163.2 ng/L and 768.3 ng/L during and after cold air exposure in the control group. Compared with Mb levels during cold air exposure, it elevated by 524.6 ng/L and 605.5 ng/L in the patient and control groups, respectively. Mb levels after cold air exposure in both the patient and control groups were significantly different compared to those before cold air exposure (*p* < 0.05). There is no significant difference in Mb levels during the same period of cold air exposure in both the patient and control groups.

#### 3.4.2. Analysis of cTnI Test Results

**Figure 2:**
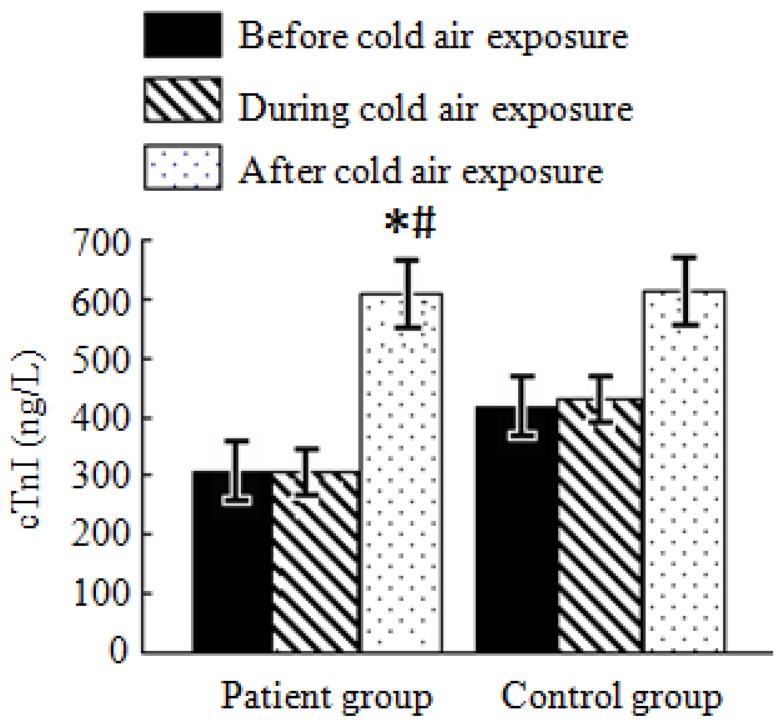
Average cTnI levels in the patient and control groups during a cold air event.

Notes: * compared with the indicators before the cold air exposure, *p* < 0.05; **#** comparison with the indicators during the cold air exposure, *p* < 0.05; cTnI: cardiac troponin I; Error bars: standard deviation.

As shown in Figure 2, cTnI levels in both the patient and control groups before and during cold air exposure increased slightly, but with no significant difference.

Compared with cTnI levels before cold air exposure, it respectively elevated by 1.2 ng/L and 4.0 ng/L in the patient and control groups during cold air exposure. Compared with cTnI levels before cold air exposure, it elevated by 306.5 ng/L and 305.3 ng/L during and after cold air exposure in the patient group while it elevated by 199.1 ng/L and 193.1 ng/L during and after the cold air exposure in the control group, respectively. CTnI levels after cold air exposure in the patient group were significantly different compared with those before and during cold air exposure (*p* < 0.05), while the difference in the control group was not significant. There is no significant difference in cTnI levels during the same cold air exposure periods in both the patient and control groups.

#### 3.4.3. Analysis of ET-1 Test Results

**Figure 3:**
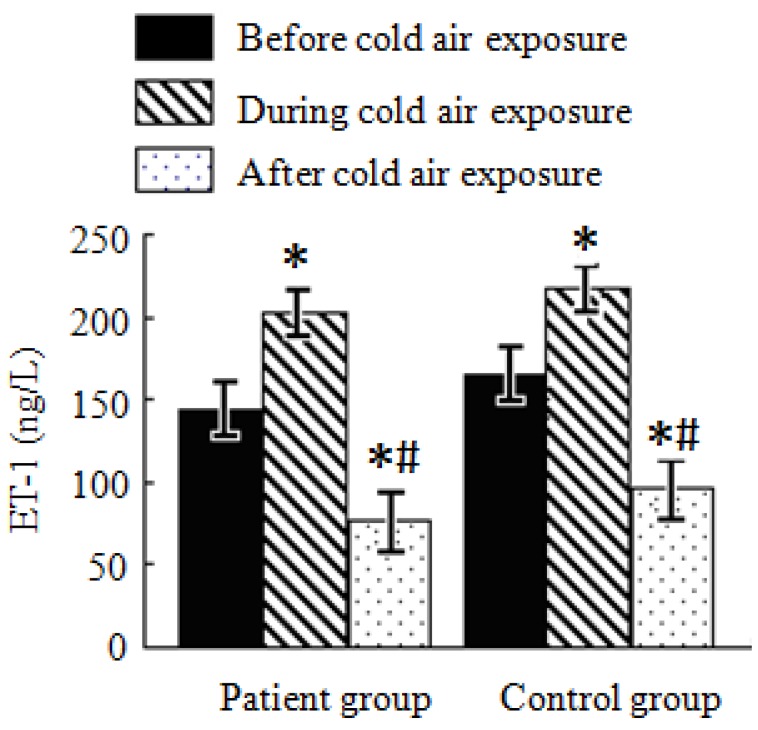
Average ET-1 levels in the patient and control groups during a cold air event.

Notes: * compared with the indicators before the cold air exposure, *p* < 0.05; **#** comparison with the indicators during the cold air exposure, *p* < 0.05; ET-1: endothelin-1; Error bars: standard deviation.

As shown in Figure 3, ET-1 levels in both the patient and control groups before, during, and after cold air exposure significantly changed (*p* < 0.05). Compared with ET-1 levels before cold air exposure, it elevated by 58 ng/L and 52.7 ng/L, respectively, in the patient and control groups during cold air exposure. ET-1 levels after cold air exposure decreased compared with levels before and during cold air exposure. In the patient group, it respectively decreased by 67.8 ng/L and 125.8 ng/L while in the control group, it respectively decreased by 69.4 ng/L and 122.1 ng/L. There are no significant changes to ET-1 levels displayed during the same cold air exposure periods in both the patient and control groups.

### 3.5. Analysis of ANG-II Test Results

**Figure 4:**
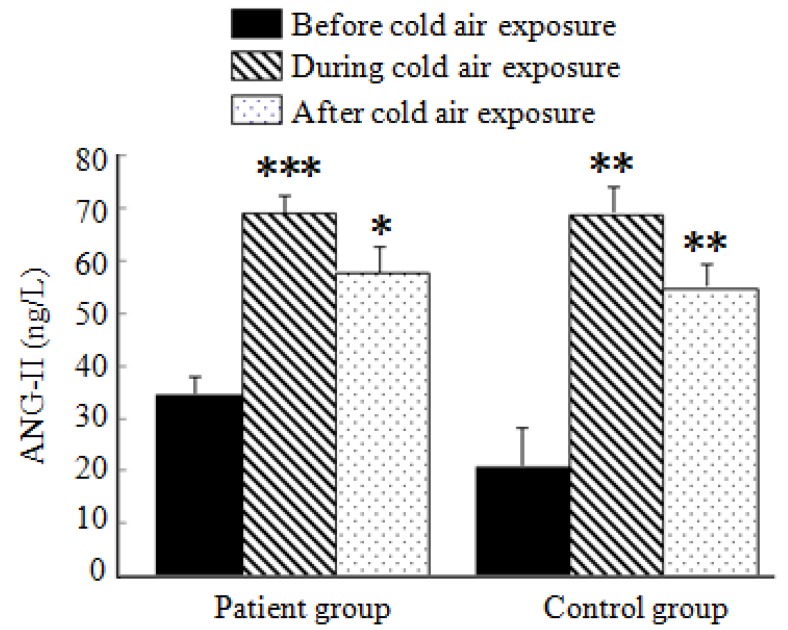
Average ANG-II levels in the patient and control groups during a cold air event.

Notes: *** compared with the indicators before the cold air exposure, *p* < 0.001; ** compared with the indicators before the cold air exposure, *p* < 0.001; * compared with the indicators before the cold air exposure, *p* < 0.005; ANG-II: angiotensin. Error bars: standard deviation.

As shown in Figure 4, ANG-II levels in both the patient and control groups before, during, and after cold air exposure increased compared with levels before the cold air exposure. ANG-II elevated by 39.1 ng/L and 46.7 ng/L during the cold air exposure in the patient and control groups, respectively; the difference was significant (*p* < 0.001). ANG-II levels in both the patient and control groups dropped significantly (*p* < 0.005). The levels lower than those observed during cold air exposure but they were still higher than those before cold air exposure (26.1 ng/L and 34.7 ng/L higher, respectively). There were no significant changes to ANG-II levels during the same cold air exposure periods in both the patient and control groups.

### 3.6. Analysis of Blood Pressure Test Results

**Figure 5:**
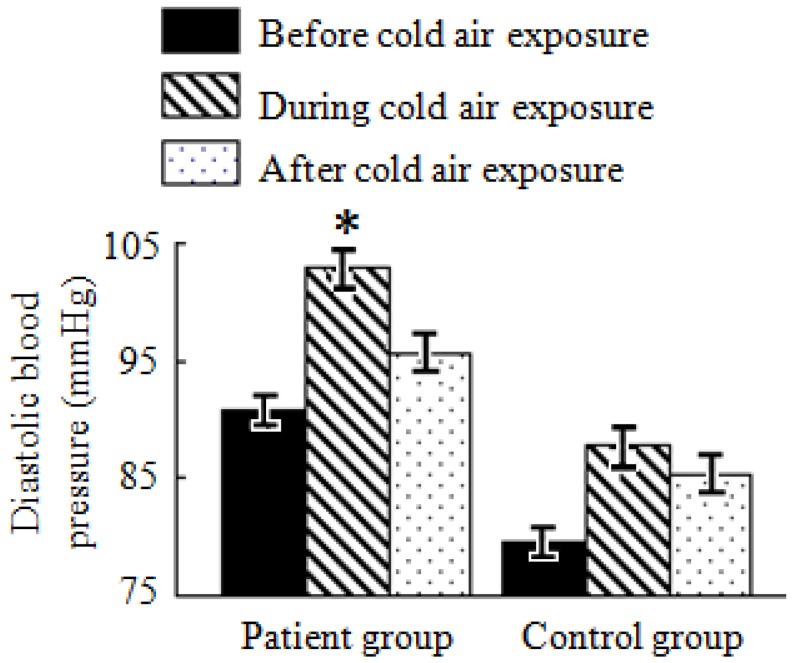
Average diastolic blood pressure levels in the patient and control groups during a cold air event.

Notes: * compared with the indicators before the cold air exposure, *p* < 0.05; Error bars: standard deviation.

As shown in Table 4 and Figure 5, systolic and diastolic blood pressure levels in both the patient and control groups increased during and after the cold air exposure compared with those before the cold air exposure. Both systolic and diastolic blood pressure levels reached the maximum value during cold air exposure. After cold air exposure, they dropped to levels lower than those during cold air exposure but still higher than those before cold air exposure. Both systolic and diastolic blood pressure levels during cold air exposure in the patient group significantly changed compared to the before cold air exposure group (*p* < 0.05); there was no significant difference in the control group. 

**Table 4:** Average systolic blood pressure levels in the patient and control groups during a cold air event (mean ± standard deviation, mmHg).

Groups	Age	Time		
		Before Cold Air Exposure	During Cold Air Exposure	After Cold Air Exposure
Patient group	40–49	121 ± 3	132 ± 5 *	127 ± 4
	50–59	124 ± 5	137 ± 6 *	133 ± 4
	60–70	129 ± 4	140 ± 7 *	135 ± 6
Control group	40–49	115 ± 5	121 ± 4	118 ± 3
	50–59	118 ± 4	127 ± 6	124 ± 4
	60–70	129 ± 5	137 ± 7	131 ± 5

Note: * compared with the indicators before the cold air exposure, *p* < 0.05.

Both the systolic and diastolic blood pressure was lower among control subjects compared with patients before, during and after cold air exposure. These results show that the cold air exposure influences both the health and the cardiovascular and cerebrovascular patients; however, the impact on cardiovascular and cerebrovascular patients is more significant.

### 3.7. Discussion

Above results showed that cold air activity could cause a significant increasing metabolism and secretion of NE, Mb, cTnI and ET-1. Also NE, Mb, cTnI still maintained a higher level of concentrations at 1 hour after the end of the cold air exposure.

To study the effects of moderate strength cold air exposure on blood pressure and biochemical indicators among cardiovascular and cerebrovascular patients, we examined blood DA, E, NE, ANG-II, Mg, cTnI, and ET-1 plasma concentration levels in both patient and control groups. NE concentration in plasma showed a trend; in the patient group, the concentration was high during and after cold air exposure. After cold air exposure, blood pressure decreased slightly, but remained at a high level. However, in the control group, it was high during cold air exposure and recovered soon after the cold air exposure. Blood pressure in the control group recovered faster than in the patient group, which also indicates that the effects of cold air on blood pressure in the patient group were longer lasting than in the healthy control group.

Elevated NE and ANG-II plasma concentrations suggest that the sympathetic nervous system (SNS) and renin-angiotensin system (RAS) were activated, respectively. Activation of these two systems will inevitably lead to elevated blood pressure. NE and ANG-II are vasoconstrictors thus they narrow the blood vessel. The effect of these two substances likely caused systemic vasoconstriction, thereby elevating the blood pressure. Many studies have fully demonstrated that an increase in blood pressure following cold air stimulation is caused by activation of the SNS and RAS [27,28]. It is found that all studies till now had focused on short-term or long-term constant low temperature stimulation by comparing the literature [29,30,31,32,33,34,35,36], which do not represent the impact of temperature changes during actual cold air weather events on the cardiovascular system. In this study focused on a cold air weather event, temperature dropped gradually, which was stimulation and this also led to NE and ANG-II increases in plasma. Therefore, we concluded that the cold air that leads to a rise in blood pressure also excited the SNS and the RAS. In addition, we found that E plasma concentration which has a strong effect on the SNS did not change significantly before, during, and after cold air exposure. A strong cold stimulus is needed to activate E responses [37]; hence, it is understandable that there was not any response observed here in a moderate strength cold air exposure. Scriven *et al.* also found that NE increased in subject groups, and E did not change significantly after cold stimulation for 30 min at 4 °C [34]. In our experiment, we also showed that NE levels increased in plasma, and there was no significant change in E levels. This means that the NE in the plasma was not secreted by the adrenal medulla, but was released from the sympathetic nerve endings [37,38]; ANG-II can also promote this pathway to increase the release of NE [39]. Therefore, the increase in NE we demonstrated with the cold air exposure was due to an increase in ANG-II. As mentioned above, cold air activity led to an increase in blood pressure in both the patient and control groups. This was mainly due to the activation of the SNS, which caused ANG-II increase and NE release, and stimulate the RAS. A combination of these two systems causes blood pressure to rise.

Mb and cTnI in serum are all indicators of myocardial injury. Mb is the only contractile protein in the myocardium, which has high sensitivity and specificity to myocardial necrosis or injury. There is a low Mb content in the blood and small amounts of myocardial necrosis will quickly elevate Mb levels [40,41]. CTnI has both a high specificity and sensitivity to myocardial injury, cTnI is currently the best marker, and is gradually becoming the standard for Acute Myocardial Infarction diagnostic criteria [42,43]. 

ET-1 in serum is an indicator of cerebral vascular injury. ET-1 is one of the most effective endogenous vasoconstrictive agents discovered and it has long lasting effects; it is endothelium-derived. ET-1 can induce intracellular calcium (Ca^2+^) overload, promote the release of excitatory amino acid neurotransmitter through its strong vasoconstrictor activity, aggravate brain tissue edema around the hematoma, and cause ischemic injury [44]. Previous studies showed that ET-1 is a humoral factor, involved in many diseases including cerebrovascular diseases. Cerebrovascular is very sensitive to ET-1, when injury occurs in this area, ET-1 levels of plasma and cerebrospinal fluid increase significantly, the sensitivity of the cerebrovascular to ET is enhanced, and ET-1 content positively correlates with severity of diseases. When cerebral infarction occurs, ET-1 formation increases in the damaged area and promotes a variety of messenger pathways by endothelin receptor activation, and promotes the release of thromboxane A_2_. As a result, there is an influx of Ca^2+^ flow resulting in intracellular Ca^2^^+^ overload, which leads to severe cerebral circulatory disturbance eventually causing brain cell death.

The above results indicated that Mb, cTnI, and ET-1—three indicators of the patient group—had varying levels of change while Mb and ET-1 in the control group showed dramatic change during and after cold air exposure. Cold air affects these indicators in both patients with cardiovascular and cerebrovascular diseases and in healthy subjects, thus affecting health via multiple factors. Analysis of results above showed that ET-1 is the most sensitive indicator and has the greatest impact on cold air exposure that affects cardiovascular and cerebrovascular diseases. 

Cold air exposure can affect Mb, cTnI, and ET-1 metabolism and secretion, seriously affecting the cardiovascular system. Our results showed that Mb levels increased during and after cold air exposure, and this increase was observed in patient and control groups. We suggest that unique meteorological conditions formed by cold air, especially when temperature and pressure change sharply in a short period, induced a stress response in the body thus increasing the blood flow in the heart and brain and blood circulation load. The increased load can damage to heart muscle cells, resulting in higher concentrations of Mb and cTnI, and this damage cannot be repaired quickly after cold air exposure. In our results, ET-1 concentration increased significantly during cold air exposure and dropped after cold air exposure to lower levels than what which was observed in before cold air exposure groups. Changes in ET-1 levels in the patient group were significant before, during, and after the cold air exposure. However, there were no significant differences in the control group before and during cold air exposure. Therefore, cold air exposure can affect ET-1 concentrations and thus affect the cerebral vascular system. Stress time, extent and results of climate environment formed by cold air activity vary by group (patients *vs. vs.* controls), so the corresponding serum indicators had varying results. Effect of cold air on the three serum indicators of the patient group was more obvious, indicating that effect of cold air on cardiovascular and cerebrovascular patients was greater than that of healthy people, probably because patients are more sensitive to cold weather. After exposure to cold air, various biochemical parameters in patients change in a short time frame, irritation conditions, and injuries specific to cardiovascular and cerebrovascular diseases conditions can easily occur. As for healthy people, there was little or no change to the serum indicators probably because the body’s ability to adapt to environmental changes [45].

Due to study limitations, there were still three main factors that could not be controlled in this study. Firstly, volunteers participant in the experiment selected from residents living near the monitoring point, were required to keep local customary ways of life without any change, however, there were still lifestyle differences among different families, and there were also differences in their working conditions, so that it was difficult to achieve exactly the same exposure history. Secondly, although a daily questionnaire for each subject was conducted to investigate and remove various confounding factors affecting the experimental results, some of such factors might still exist. Thirdly, blood pressure measurement results might be confounded by large age span of the volunteers; in addition, the results were influenced by progresses of the diseases in different volunteers of patients group. More perfect experiments would be designed in future to reduce or avoid the uncertainties as much as possible.

## 4. Conclusions

Based on our results, we can draw the following conclusions: (1) Considering the influence of cold air exposure on cardiovascular and cerebrovascular diseases, ET-1, DA and, ANG-II are the most sensitive indices, and have the greatest impact. (2) The pathway that cold air exposure led to an increase in blood pressure in cardiovascular disease patients and healthy subjects is the activation of the SNS, which caused ANG-II increase and NE release, and stimulate the RAS. The combined effect of these systems led to a rise in blood pressure. The impact of cold air exposure on the change of blood pressure in cardiovascular patients was more significant than in healthy people, and the effect on the cardiovascular patients lasted longer. (3) Cold air can cause significant metabolism and secretion of Mb and cTnI; Mb and cTnI remained at relatively high levels at the end of the cold air exposure. This showed that the cold air exposure can cause damage to human heart cells, and the damage is not repaired quickly. (4) Metabolism and secretion of ET-1 increased significantly during cold air exposure, and dropped after cold air exposure to lower levels than that before cold air exposure. This indicated that the cold air exposure can cause damage to human brain cells, but the damage is repaired quickly.
